# An Automatic Clock-Induced-Spurs Detector Based on Energy Detection for Direct Digital Frequency Synthesizer

**DOI:** 10.3390/s22093396

**Published:** 2022-04-28

**Authors:** Xin Lei, Junan Zhang, Jun Deng, Peng Yin, Zhou Shu, Fang Tang

**Affiliations:** 1 School of Microelectronics and Communication Engineering, Chongqing University (CQU), Chongqing 400044, China; leixin2003@cqu.edu.cn (X.L.); dj_richard@cqu.edu.cn (J.D.); yinpeng9527@cqu.edu.cn (P.Y.); shuzhou@cqu.edu.cn (Z.S.); 2 College of Artificial Intelligence, Chongqing University of Technology, Chongqing 400054, China; zja2017@cqut.edu.cn

**Keywords:** spur detector, programmable low-pass filter, direct digital frequency synthesizer, spur-detection algorithm

## Abstract

A clock-induced-spurs detector, composed of a programmable low-pass filter (LPF), energy detector and spur detection algorithm, is presented and applied to a four-channel 1 gigabit-samples-per-second (GSPS) direct digital frequency synthesizer (DDS). The proposed detector realizes the detection of spurs based on energy-detection, and the spur detection algorithm is adopted to automatically extract the amplitude and phase of clock-induced spurs, generated by the intermodulation of harmonic spurs and multiple clocks. Finally, the extracted features are sent to auxiliary DDS to decrease the target spur, following which the detector can be turned off to save power. Additionally, the detected characteristics under different output conditions can be read out through the interface for rapid frequency switching. The proposed detector integrated into a DDS is fabricated with a 65 nm complementary metal oxide semiconductor (CMOS) process and has an area of 190 μm × 320 μm. The measured power consumption is roughly 38 mW, consuming 6% that of a single-channel DDS. The test results show that the spurious-free dynamic range (SFDR) of this DDS can be successfully enhanced from −43.1 dBc to roughly −59.9 dBc without any off-chip instruments. This effectively proves that the detection accuracy of this detector can reach around −81 dBm.

## 1. Introduction

**Figure 1:**
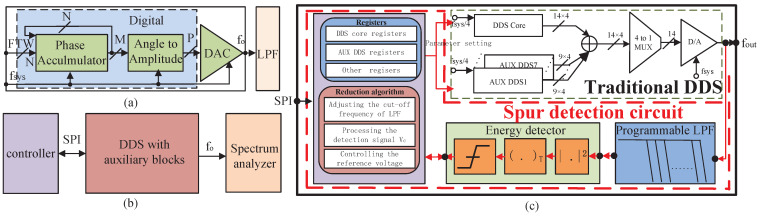
(**a**) The traditional architecture, (**b**) the traditional output spectrum reduction system, and (**c**) the spurs detection system of traditional DDS.

With the development of digital signal processing and integrated circuit technology, the direct digital frequency synthesizer (DDS) has become a popular solution for clock and signal generation, frequency modulation and test equipment [1,2]. Figure 1a shows the block diagram of a traditional DDS, in which the DDS core, that is composed of an N-bit phase accumulator and an angle-to-amplitude converter, is followed by a digital-to-analog converter (DAC). The output frequency $f_{o}$ is proportional to the frequency turning word (FTW), as shown in (1), where $f_{sys}$ is the system clock and N is the quantized digits of the phase accumulator [3]. In addition, the amplitude scale factor (ASF) and phase offset word (POW) are used to set the amplitude and phase of DDS, respectively.

(1) $$\begin{array}{c} f_{o}=\frac{\mathrm{FTW}}{2^{N}}f_{sys}\;\;\;\;\;\;\;\;\;\;\;\;\;\;\;\;\;\;\;\;\;\;\;\;\;\;\;\;\;\;\;\;\;\;\;\;\;\;\;\;0\leq \mathrm{FTW}\leq 2^{N-1} \end{array}$$

(2) $$\begin{array}{c} f_{p}=A\cdot \frac{f_{sys}}{B}\pm C\cdot f_{o}\;\;\;\;\left(B=1,2,4...A,C=1,2,3...\right) \end{array}$$

The spurious-free dynamic range (SFDR) which is limited by the maximum spurs, is a key index of DDS [4]. There are multiple reasons that spurs exist, including the amplitude quantization [5], the truncating of the phase accumulator [6], digital-to-analog (D/A) conversion error and so on. The most significant spurs are usually generated during D/A conversion, mainly due to the non-linearity of DAC, the non-ideal characteristics of switches and the quantization error [7,8,9]. Meanwhile, the time-interleaved architecture is adopted by the high-speed DDS to make its digital blocks operate in a lower frequency, which inevitably introduces multiple clock frequencies. These clocks and harmonic spurs modulate each other through substrate, power, and ground coupling to produce a series of frequency-determined spurs known as clock-induced-spurs, which are the most decisive factor for SFDR. The location of these spurs can be described in (2), where $f_{o}$ and $f_{p}$ are the output frequency and spurs’ frequencies, respectively [10]. The authors in [9,10] present a method for adding auxiliary DDS blocks to reduce clock-induced-spurs for DDS. However, for different output frequencies, the spurs characteristics will change synchronously, and it is difficult to estimate them accurately. So, external equipment is required to obtain the characteristics of these spurs, which significantly limits the convenience of this method, as shown in Figure 1b. This paper proposes a clock-induced-spur detector based on energy-detection technology, with relatively simple implementation and calculation [11], which can be used for extracting the characteristics of spurs. We applied it to a 1 gigabit-samples-per-second (GSPS) DDS. The contributions of this article are as follows. Firstly, a fourth-order low-pass filter (LPF) with 63 programmable cut-off frequencies between 11 megahertz (MHz) and 500 MHz is constructed to cover the different spurs. Then, based on the principle of energy-detection, the square operation of the spurs is adopted to detect their direct-current (DC) component. The energy-detection circuit and algorithm cooperate to realize the perception of spurs existence, further extract the amplitude and phase features of the spurs, and provide them to the auxiliary DDSs for spurs reduction. Combined with the algorithm, the amplitude and phase of the spurs are then detected and provided to auxiliary DDS for spur reduction. Then, these detected features can be read out through a serial peripheral interface (SPI) with a simple design, wide application and relatively high transmission rate, thus meeting the needs of rapid switching between different output frequencies. Compared with the DDS block, this spurs detection circuit only consumes a small proportion of the power and area. This 14-bit DDS adopting the proposed spurs-detection circuit, working under a 1 gigahertz (GHz) clock, is fabricated using a standard 65 nm complementary metal oxide semiconductor (CMOS) process whose largest output frequency is 400 MHz. The spur detector can improve the SFDR to about −59.9 dBc, and this effect is proven by measurement results. Importantly, compared with some DDS using external equipment, the DDS adopting the proposed spurs-detection method has the potential for wider application in industry. The remainder of this paper is organized as follows: Section 2 introduces the system architecture of the proposed spur detection circuit, and Section 3 shows the algorithm and key circuits of the detector. Section 4 presents the experimental results and the comparison, and finally, the conclusion is given in Section 5.

## 2. System Architecture

Figure 1c shows the architecture of the chip integrating the proposed spurs-detector and the traditional DDS with auxiliary DDSs, which is controlled by SPI. The chip has a DDS core with a 32-bit frequency-tuning word [12], thus it meets the frequency–accuracy requirements of the mainstream commercial products, and it adopts a time-interleaved architecture to enable digital blocks to operate in 250 MHz ($f_{sys}$/4) before being mixed to a 14-bit linear current-steering DAC. Among them, the 250 MHz digital clock frequency is evaluated according to the upper limit frequency of the digital library and determined by the compromise between working frequency, circuit area, power consumption and complexity. On the other hand, the quantized bits of DAC are the result of a trade-off between noise floor, output signal dynamic range, area and power consumption. The auxiliary DDS core blocks (AUX DDS1–AUX DDS7) are used for spurs-reduction, and their operation principle is as follows. Suppose the target spur is Asinωt, where ω and A represent the frequency and amplitude, respectively. Thus, the spur generated by the auxiliary DDS core is $\left(A+\Delta A\right)\sin\left(\left(\omega +\Delta \omega \right)t+\pi +\Delta \theta \right)$, in which, the Δω, ΔA, and Δθ are the deviations of frequency, phase, and amplitude from the ideal value. Equation (3) shows the remaining error $E_{r}$ after adding the target and auxiliary spurs together. Thus, clearly, if Δω, ΔA, and Δθ are small enough, the approximate equation $E_{r}\approx A\sin\omega t+A\sin\left(\omega t+\pi \right)$ can be used to reduce the spurs significantly. Once the frequencies of clock-induced spurs are determined, spurs can be reduced just by setting the ASF and POW of the auxiliary DDS core, respectively. Based on this principle, the reduction operation can be carried out once the characteristic of the spur is detected. To detect a spur, the first step is to understand its coverage by programming the pass-band of LPF. Then, the energy detector detects the presence of said spurs. If the spur is detected, one auxiliary DDS will be turned on and its output will synchronously be superimposed onto the target spur. Then, the proposed algorithm will simultaneously scan the ASF and POW of the auxiliary DDS. After each scanning operation, the size of the residual error $E_{r}$ should be detected by the energy detector and compared with the output of an adjustable reference circuit (ARC) to deliver the detection decision. The algorithm will make a judgment and decide whether or not to continue scanning until the spur is reduced to the expected value.

(3) $$\begin{array}{c} \begin{array}{c} E_{r}\approx A\sin\omega t+\left(A+\Delta A\right)\sin\left(\left(\omega +\Delta \omega \right)t+\pi +\Delta \theta \right) \end{array} \end{array}$$

**Figure 2:**
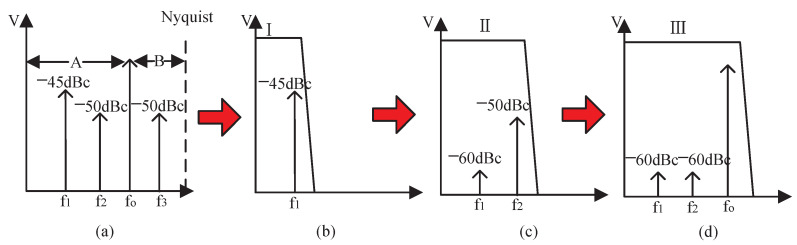
Operating flow of spur detection and reduction: (**a**) dividing the frequency by $f_{o}$, (**b**) filtering and spur detection of $f_{1}$, (**c**) reduction of $f_{1}$ and detection of $f_{2}$, (**d**) reduction of $f_{2}$ and remove $f_{3}$ by off-chip filter.

Figure 2 shows the the operation flow of spur reduction based on the proposed spur detector, in which $f_{o}$ is the output frequency of DDS, and there are three spurs $f_{1}$–$f_{3}$, bigger than −60 dBc between DC and Nyquist frequency. The frequency band is divided into two parts by $f_{o}$; those lower than and higher than $f_{o}$ are bands A and B, respectively. Because the spurs in band B can be attenuated by the off-chip LPF, this paper mainly focuses on the spurs-reduction for band A. As shown in Figure 2b, firstly the detection circuit controls the pass-band width of LPF to ensure that $f_{1}$ is located in the pass-band. Secondly, an immediate detection of spurs by the energy detector in the pass-band enables the spurs’ amplitude and phase to be extracted through the corresponding algorithm. Finally, $f_{1}$ can be reduced to about −60 dBc by setting the auxiliary DDS with the extracted features, then the reduction block continues for the remaining spurs. Figure 2c shows the reduction of a spur at $f_{2}$, which is similar to that described in Figure 2b. Finally, the DDS reduces the spurs $f_{1}$ and $f_{2}$ all to about −60 dBc, as indicated in Figure 2d. Thus, the off-chip LPF can filter out the spurs in band-B.

## 3. Implementation of Spur Detection

### 3.1. Energy Detector

In order to extract the target spurs, the existence of the spur needs to be confirmed. Energy-detection technology can measure the energy of the signal and compare it with the threshold in order to detect the signal, which can be well-realized by integrating the square and comparator [13]. The $E_{r}$ shown in (3) will be processed as an input signal to the proposed energy detector. Firstly, since the clock-induced spurs that need to be detected are frequency-determined, the frequency deviation is small enough to make Δω=0. Then, expanding the $E_{r}$ given in (3) and removing the second-order multiplication term can allow us to obtain its approximate value, shown as (4), in which $\tan\varphi =\Delta A/\left(-A\cdot \Delta \theta \right)$.

(4) $$\begin{array}{c} E_{r}\approx -A\cos\omega t\cdot \Delta \theta +\Delta A\sin\omega t=\sqrt{A^{2}\cdot \Delta \theta^{2}+\Delta A^{2}}\sin\left(\omega t+\varphi \right) \end{array}$$

$E_{r}$ can be squared in (5), where $A_{s}=\sqrt{A^{2}\Delta \theta^{2}+\Delta A^{2}}$. Obviously, one LPF can filter out its high-frequency components based on retaining the DC components.

(5) $$\begin{array}{c} \begin{array}{c} S_{s}=E_{r}^{2}=\frac{A_{s}^{2}}{2}-\frac{A_{s}^{2}}{2}\cdot \cos\left(2\omega t+2\varphi \right) \end{array} \end{array}$$

**Figure 3:**
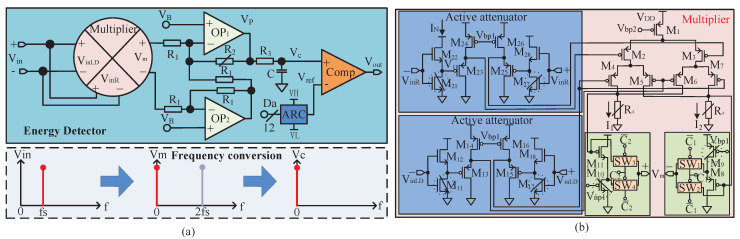
(**a**) Circuit structure of the energy detector, (**b**) circuit of the multiplier.

The spur detector is shown in Figure 3a, and mainly consists of a multiplier, a differential signal amplification circuit, an LPF and a comparator. The multiplier realizes the square operation of a spur and converts it into DC and a higher frequency. Subsequently, the differential signal-amplification circuit based on the operational amplifier (OP) is adopted to amplify the squared spur and then an R-C LPF is used to filter out the high-frequency components. Finally, the amplified signal is compared with the output of ARC; if the comparator shows that $V_{c}$ is greater than $V_{ref}$, the existence of spurs is suggested. The multiplier shown in Figure 3b adopts a Gilberto structure [14], and an active attenuation circuit is added at the input of the multiplier to obtain a wide input range. Taking the input port $V_{inR}-$ as an example, the input signal is attenuated through a circuit composed of M21 and M22, which work in the linear region and saturation region, respectively. Since the currents $I_{N}$ flowing through M21 and M22 are equal, Equation (6) can be obtained, where $\left(W/L\right)$ is the width to length ratio of the corresponding transistor, $K_{N}=1/2\mu_{n}C_{ox}$, in which $\mu_{n}$ and $C_{ox}$ are the mobility of the electronics in the inversion layer and the oxide capacitance per unit area, respectively.

(6) $$\begin{array}{c} \begin{array}{c} K_{N}{\frac{W}{L}}_{21}\left[2\left(V_{inR+}-V_{1}-V_{TH21}\right)V_{DS21}-V_{DS21}^{2}\right]\\ =K_{N}{\frac{W}{L}}_{22}{\left(V_{inR+}-V_{TH21}\right)}^{2} \end{array} \end{array}$$

Putting the $V_{DS21}=V_{DD}-V_{1}$ into (6), we obtain the node voltage $V_{1}$ as shown in Equation (7). Naturally, the attenuation of $V_{inR}-$ can be realized by changing ${\left(W/L\right)}_{21}$ or ${\left(W/L\right)}_{22}$, and finally the voltage of $V_{1}$ will be shifted by M23 and input to the multiplier.

(7) $$\begin{array}{c} V_{1}=\left(1-\sqrt{\frac{{\left(W/L\right)}_{21}}{{\left(W/L\right)}_{21}+{\left(W/L\right)}_{22}}}\right)\left(V_{in}-V_{TN}-V_{DD}\right)+V_{DD} \end{array}$$

Equation (8) shows the output voltage $V_{m}$ of the multiplier when the input spur $V_{in}$ is Asinωt, where $A_{1}$ is the gain of the multiplier and $K_{P}=1/2\mu_{p}C_{ox}$. ${\left(W/L\right)}_{a}$ is the width-to-length ratio of $M_{2}$ and $M_{3}$, and the ${\left(W/L\right)}_{b}$ is the width-to-length ratio of $M_{4}$–$M_{7}$.

(8) $$\begin{array}{c} V_{m}=K_{P}\sqrt{\frac{1}{2}{\left(\frac{W}{L}\right)}_{a}{\left(\frac{W}{L}\right)}_{b}}A_{1}A^{2}\left(\cos2\omega t+1\right)R_{4} \end{array}$$

Then, the $V_{m}$ will be amplified by amplifiers $OP_{1}$ and $OP_{2}$ to $V_{P}$ shown in (9), and its amplification factor is determined by the ratio of resistors $R_{2}$ and $R_{1}$. Meanwhile, load capacitance C and resistor $R_{3}$ of OP form the main poles $1/\left(R_{3}C\right)$ of the filter, which can attenuate the high-frequency components of the spur. In addition, considering the non-ideal characteristics of the filter transmission band, there may be other spurs or a residual output signal in the pass-band when reducing the target spur. After the square operation of the multiplier, all spurs are transmitted to the subsequent differential amplifier circuit in the form of a DC component. If the DC components generated by these signals are too large, the differential amplifier circuit may be saturated. Therefore, a level shift circuit is added at the output of the multiplier to reduce the residual DC component. For example, at the negative output port of the multiplier, the complementary signals $C_{1}$ and $\bar{C_{1}}$ can control the switches $SW_{1}$ and $SW_{2}$ to determine whether to output from the drain or the source of M8. If choosing the source, the residual DC component can be reduced by a gate source voltage of M8, which can be adjusted by changing the width to length ratio of M9.

(9) $$\begin{array}{c} V_{P}=V_{m}\frac{R_{2}}{R_{1}}+V_{B} \end{array}$$

Finally, the filtered voltage $V_{c}$ will be compared with the reference voltage $V_{ref}$. If higher than $V_{ref}$, this indicates the existence of spurs. In order to realize the automatic detection of spurs, $V_{ref}$ must be controlled by digital signals. Therefore, a low-power 12-bit DAC based on shared resistance string architecture [15] is used to realize the ARC. The ARC can adjust the reference voltage $V_{ref}$ according to the input 12-bit digital codes, Da, and its conversion equation is shown in (10). Among them, $D_{a1}$ to $D_{a12}$ are the 12-bit control codes output by digital circuits, and VH and VL are high and low reference voltages provided outside the chip, respectively. In application, the output voltage $V_{c}$ of the amplifier must be located between VH and VL, so the approximation of $V_{ref}$ to $V_{c}$ and the adjustment of the comparison voltage can be realized according to the digital algorithm.

(10) $$\begin{array}{c} V_{ref}=\left(VH-VL\right)\left(\frac{D_{a1}}{2}+\frac{D_{a2}}{2^{2}}+\cdots +\frac{D_{a12}}{2^{12}}\right) \end{array}$$

On the other hand, the comparator adopts a dynamic architecture to effectively reduce the power and area overhead [16], and the accuracy of spur detection is determined by the comparator, the ARC and the previous $V_{c}$ generation circuit combined. Assuming that the spurs before and after reduction are $A_{a}\sin\left(\omega_{a}t+\theta_{a}\right)$ and $A_{b}\sin\left(\omega_{a}t+\theta_{b}\right)$, respectively, then the voltage difference $\Delta V_{c}$ generated at the comparison port is $1/2KA_{1}A_{2}\left(A_{a}^{2}-A_{b}^{2}\right)$. If $\Delta V_{c}$ is less than the resolution of the detector, spur-reduction cannot be realized. What should to be pointed out is that voltage offsets happen in all blocks due to process variation, but they cannot affect the accuracy of the spur detection. Equation (11) shows the equivalent offset voltage $V_{os}$ at the input of the comparator, where the $A_{1}$, $A_{2}$ and $V_{\mathrm{os}1}$, $V_{os2}$ are the gain and offset voltage of the mixer and amplification unit, respectively, and $V_{os3}$ is the offset voltage of the comparator. This will eventually be cancelled out by the DC voltage generated by ARC.

**Figure 4:**
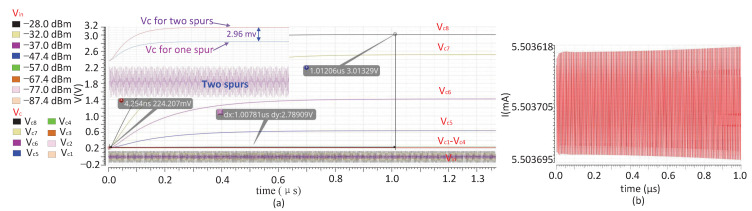
The simulation of the spur detector: (**a**) output signal $V_{c}$ of the LPF corresponds to the input spurs $V_{in}$ of different amplitudes, (**b**) dynamic operating current when the frequency of $V_{in}$ is about 400 Mhz.

The simulation result in Figure 4a shows that when the amplitude of the input spur $V_{in}$ at 400 MHz changes between −87.4 dBm and −28.0 dBm, the corresponding output voltage $V_{c}$ of the LPF varies from 0.224 V to 3.013 V, and the response time of the detector is about 1 μs. On the other hand, when detecting two spurs with different frequencies and amplitudes of −67 dBm, plus when a single −67 dBm spur is also simulated, the detector shows that the difference in output voltage $V_{c}$ is about 2.96 mv, which can be used to reflect the reduction of spurs. Figure 4b shows the simulation results of the operating current, where the dynamic current is about 5.5 mA. This current is only generated when spurs-detection is performed, and it can be turned off to optimize power consumption after setting the auxiliary DDS.

(11) $$\begin{array}{c} V_{os}=\left(V_{os1}\cdot A_{1}+V_{os2}\right)\cdot A{}_{2}+V_{os3} \end{array}$$

### 3.2. Spurs Detection Algorithm

**Figure 5:**
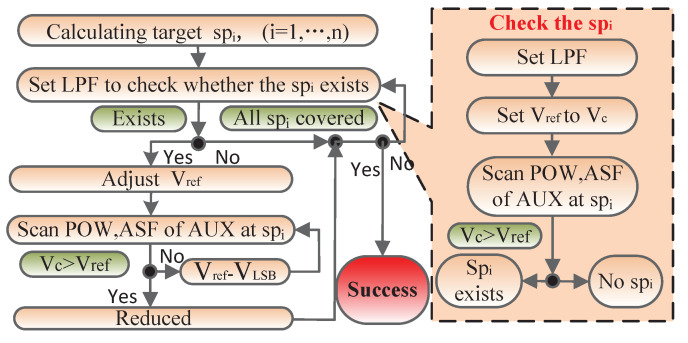
Algorithm flow of the proposed spur detection circuit.

Accurately detecting the phase and amplitude of spurs can be realized by combining the spur detection algorithm with the detector introduced above. First, the algorithm shown in Figure 5 calculates the clock-induced spurs with Equation (2), and then selects the spurs which are lower than the output frequency $f_{o}$ as target points. In fact, not all the points produce large spurs, thus, all the target spurs must be checked. The checking operation is carried out by setting the pass-band of the programmable LPF to cover the lowest target spur $sp_{1}$, after which the output of the LPF is sent to the spur detector introduced above to generate the detection signal $V_{c}$. The $V_{c}$ may include the DC component of the target spur and other signals, such as other spurs or the residue of output signals due to the non-ideal filter. Assuming that the target spur and other signals are $A_{s}\sin\left(\omega_{1}t+\theta_{1}\right)$ and $A_{r}\sin\left(\omega_{2}t+\theta_{2}\right)$, respectively, the Vc voltage can be expressed by $V_{m}=K_{P}\sqrt{1/2{\left(W/L\right)}_{a}{\left(W/L\right)}_{b}}A_{1}\left(A_{s}^{2}+A_{r}^{2}\right)$ according to (8). Then, the reference voltage $V_{ref}$ can be controlled by ARC to approach the $V_{c}$ through successive approximation logic. On this basis, $V_{c}$ can be changed by scanning the amplitude and phase of the auxiliary DDS, meaning that if the target spur exists, the $V_{c}$ will finally decrease; otherwise, it will always increase. In this way, we can detect whether the target spur exists by observing the output of the comparator. When the target spur is detected, the “Adjust $V_{\mathrm{ref}}$” operation shall be performed, which aims to reduce $V_{ref}$ through ARC. The reduced value can be controlled by the register, whose size must not to exceed the upper limit of the accuracy of the spur detector. Then, scanning the phase and amplitude of the auxiliary DDS and observing whether the output of the amplifier is reversed can effectively reduce the target spur. In order to achieve the optimal detection effect, it is necessary to reduce $V_{ref}$ by one least significant bit of ARC after each detection operation, and then to repeat the operation until no further detection is possible. Once the $sp_{1}$ detection process is completed, the operation for the next spur which is similar to $sp_{1}$ will start. This continues until the algorithm detects all of the target spurs.

It should be pointed out that the total scanning time is the key index of this algorithm, and the spur detector consumes the greatest amount of time due to the LPF. Equation (12) shows the time $T_{s}$ for scanning the amplitudes and phases of a target spur, in which, the $t_{s}$ is the time needed for one energy-detection operation, $A_{t}$ is the amplitude of the target spur, $\Delta A_{t}$ and $\Delta \theta_{t}$ are the amplitude and phase difference between the initial value of auxiliary DDS and the target spur, respectively, and *M* and *N* are the quantization bits of the phase and amplitude of auxiliary spurs, respectively. Naturally, the scanning time $T_{s}$ is proportional to $\Delta A_{t}$, $\Delta \theta_{t}$, *M* and *N*.

(12) $$\begin{array}{c} T_{s}=\frac{\Delta A_{t}}{A_{t}}\cdot 2^{N}\cdot \frac{\Delta \theta_{t}}{2\pi }\cdot 2^{M}\cdot t_{s} \end{array}$$

**Figure 6:**
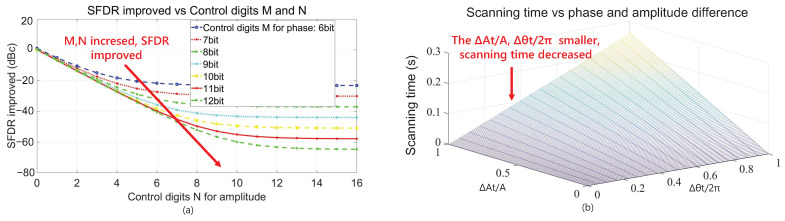
(**a**) Influences of the magnitude and phase quantization bits on SFDR, (**b**) The scanning time vs. $\Delta A_{t}$ and $\Delta \theta_{t}$.

In order to determine the appropriate values of *M* and *N*, the ΔA and Δθ are quantified in (4) with $\Delta \theta =2\pi /2^{M}$ and $\Delta A=A/2^{N}$, respectively. On this basis, the relationship between improved SFDR and *M*, *N* can be obtained by carrying out a logarithm operation on $E_{r}$ after quantification. Figure 6a shows the simulation results of the relationship between the growth in SFDR and quantized digits *M*, *N*. Considering trade-offs between improved SFDR, circuit scale and scanning time, this algorithm chooses both *M* and *N* to be 9, thus the SFDR can be reduced by up to −43 dBc. Figure 6b shows the relationship between scanning time and $\Delta A_{t}/A$, $\Delta \theta_{t}/2\pi$ when setting the $t_{s}$ to an approximate value of 1 μs. Theoretically, the maximum scanning time is about 259 microseconds for one target spur when $\Delta A_{t}/A$ and $\Delta \theta_{t}/2\pi$ are both 1. In addition, the detected information can be read out through SPI. According to corresponding output frequencies, they can be called directly to meet the requirement for rapid switching between different output frequencies.

### 3.3. Programmable Low-Pass Filter

**Figure 7:**
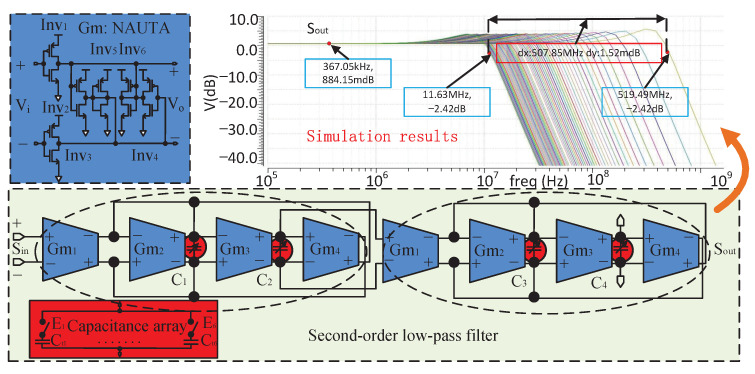
Circuit structure and simulation results of the fourth-order programmable filter.

In order to sense spurs, a pass-band width up to hundreds of megahertz of LPF must be programmable. The LPF needs to change the cut-off frequency with the frequency of output signals according to the digital algorithm, so as to attenuate the high-frequency spurs and the output signal as much as possible when detecting the target spur. The more programmable the cut-off frequencies, the higher the accuracy of the frequency band divided by LPF. However, it will increase the power consumption and area. During the design stage, we carefully considered the trade-off between frequency band division accuracy and chip resource overhead, and finally adopted 63 programmable cut-off frequencies. In this way, we can ensure that each frequency point in the target frequency band can be covered by at least one −3 dBc bandwidth of the programmable LPF, thus ensuring good tracking and attenuation effects for different output signals. Figure 7 shows the programmable fourth-order filter which is composed of two second-order filters in series with a capacitor array. Equation (13) shows the transfer function of the second-order filter, in which ${gm}_{1}$–${gm}_{4}$ are the transconductances of the amplifiers ${Gm}_{1}$–${Gm}_{4}$, while the $C_{1}$ and $C_{2}$ are the programmable capacitances. The DC gain $A_{0}$ and cut-off frequency $\omega_{0}$ of the second-order filters are $gm_{1}/gm_{4}$ and $\sqrt{gm_{3}\cdot gm_{4}/\left(C_{1}\cdot C_{2}\right)}$, respectively. Naturally, the numerical control of the pass-band can be realized by programming the capacitor array. The chip integrates six binary-weighted capacitors $C_{t1}$–$C_{t6}$ to realize 63 capacitor values of $C_{1}$ and $C_{2}$, while the complementary switches control the size of the capacitor array. Because the spur detection only focuses on the change value of the output signal energy of the fourth-order filter, there are no special requirements for common indicators such as ripple, phase linearity and pass-band flatness of the filter. For the fourth-order filter, the slope of the transition band is the key index, the steeper it is, the better the attenuation effect of the programmable filter on the output signal is. However, in this case, more programmable cut-off frequencies are required to meet the demand that all frequency points of the target frequency band are located within the −3 dB bandwidth corresponding to at least one cut-off frequency, which brings greater overhead. Therefore, the slope of the transition band needs to be considered in combination with the number of programmable cut-off frequencies. Equation (13) shows that transition band width changes with the different values of $C_{1}$ and $C_{2}$, in particular, the values get bigger and the band gets narrower.

(13) $$\begin{array}{c} \frac{V_{out}\left(s\right)}{V_{in}\left(s\right)}=\frac{gm_{1}\cdot gm_{3}/\left(C_{1}\cdot C_{2}\right)}{s^{2}+s\cdot gm{}_{2}/C_{1}+gm_{3}\cdot gm_{4}/\left(C_{1}\cdot C_{2}\right)} \end{array}$$

The Gm units are all the Nauta amplifiers [17] made up of four inverter-amplifier units ${Inv}_{1}$–${Inv}_{4}$ with high speed. The ${Inv}_{1}$ and ${Inv}_{2}$ are the differential input terminals which provide gain, and are followed by the ${Inv}_{3}$ and ${Inv}_{4}$ to form a negative impedance loop. In the meantime, ${Inv}_{5}$ and ${Inv}_{6}$ ensure common-mode feedback to provide DC bias to the output port. The simulation results show that the pass-band of the LPF covers from about 11 MHz to 507 MHz.

### 3.4. Noise Performance and Monte-Carlo Analysis

The spur detection circuit, composed of the programmable LPF and spur detector shown in Figure 1c, has noise characteristics which are mainly affected by high-frequency thermal noise and flicker noise. Because the LPF is located in the first stage of the spur-detection circuit, its noise performance becomes the key factor affecting the overall noise characteristics. First, the high-frequency output noise of the Nauta unit shown in Figure 7 can be expressed via (14), in which $gm_{i}$ are the transconductances of the six inverters and c is their thermal noise coefficient of [17]. The resulting equivalent input noise can be expressed as ${\bar{V}}_{in}^{2}={\bar{i}}_{od}^{2}/g_{ma}^{2}$, where $g_{ma}$ is the equivalent transconductance of the Nauta unit.

(14) $$\begin{array}{c} {\bar{i}}_{od}^{2}=4kTc\Delta f\sum gm_{i} \end{array}$$

**Figure 8:**
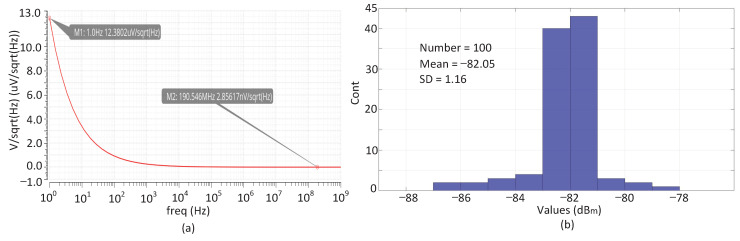
(**a**) Equivalent input noise of spur detection circuit, (**b**) Results of Monte-Carlo simulation for detection accuracy.

On the other hand, the noise of the circuit after the LPF can also be converted to the input. For example, the high-frequency output noise of the mixer can be expressed through (15), in which γ, A, I and $R_{L}$ are the channel noise factor, local oscillator amplitude, mixer output amplitude and load resistance, respectively [18]. The resulting equivalent input noise can be expressed as ${\bar{V}}_{inm}^{2}={\bar{V}}_{od}^{2}/\left(A_{va}^{2}A_{vd}^{2}\right)$, where $A_{va}$ and $A_{vd}$ are the amplitude gains of the Nauta unit and multiplier, respectively. By properly designing transconductance $g_{ma}$, a compromise can be found between noise and technical indexes such as power and area. Finally, according to the requirements of flicker noise, we use the p-channel metal oxide semiconductor as input, and the load resistances of the mixer are free of flicker noise. Figure 8a shows the simulation results of the equivalent input noise of the spur-detection circuit. The maximum equivalent input noise density located in the low-frequency region is about 12 μV/Hz, and the equivalent integral input noise between 1 Hz and 1 GHz is about 96 μV. At the same time, due to the limitations regarding the accuracy of the spur-detection circuit described above, the minimum spur that can be processed is roughly −60 dBc, meaning that its amplitude is about 1 mV. Therefore, the noise characteristics of this circuit can meet the requirements of the minimum spurs processing. The simulation results of 100 Monte-Carlo iterations are shown in Figure 8b, where the average detection accuracy and standard deviation (SD) are −82.05 dBm and 1.16 dBm, respectively.

(15) $$\begin{array}{c} {\bar{V}}_{on}^{2}=8kTR_{L}\left(1+2\gamma R_{L}I/\pi A+\gamma g_{m}R_{L}\right) \end{array}$$

## 4. Measurement Results and Comparison

**Figure 9:**
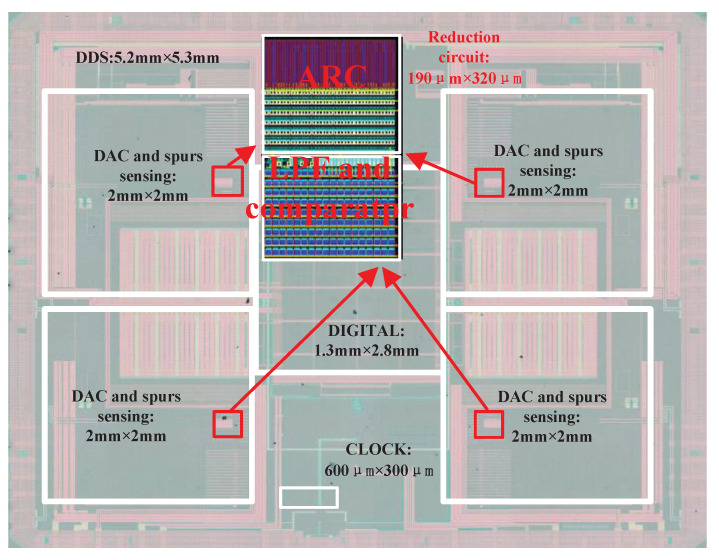
Chip microphotograph of the DDS.

This paper reports a spur detector that was applied to a four-channel DDS for the application of multiple channel communication systems on a 1-GHz clock. The chip was fabricated using a 65 nm CMOS process with a 27.6 mm^2^ area, in which the detection circuit accounts for only 190 μm × 320 μm of the area shown in Figure 9.

**Figure 10:**
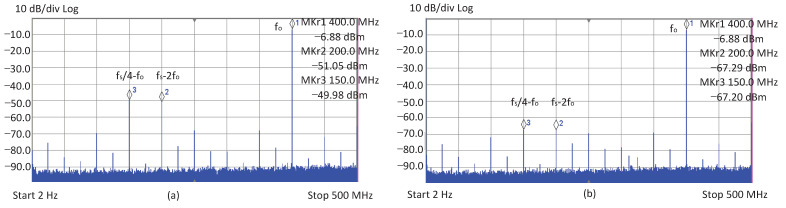
The spectrum of 400 MHz output: (**a**) Spectrum of before spurs reduction, (**b**) Spectrum of after spurs reduction.

**Figure 11:**
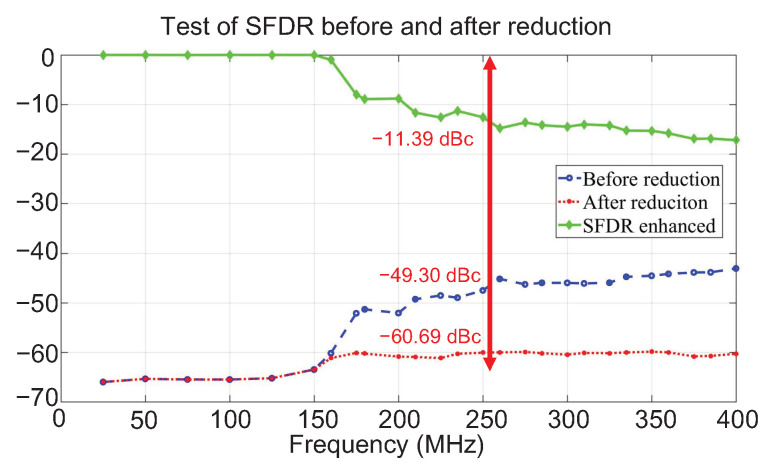
The enhancement of SFDR at different output frequencies.

The test system mainly consisted of the design-under-test (DUT) chip, MG3691A signal generator, IT6332B DC power supply and R&S FSUP signal source analyzer. The test results are shown in Figure 10a, where the $f_{o}$ is set to the maximum value of 400 MHz, there are two spurs larger than −50 dBc, and they are all clock-induced-spurs located in $f_{s}/4-f_{o}$ and $f_{s}-2f_{o}$, respectively. Figure 10b indicates that the two spurs can be successfully detected by the detector, and can be reduced to enhance the SFDR from around −43.10 dBc to −60.32 dBc. Figure 11 shows the test results of the SFDR before and after reduction at multiple output frequency points from DC to 400 MHz. It can be seen that when the output frequency exceeds 150 MHz, the proposed detector starts to work and increases the SFDR index to about −60 dBc. However, when the output frequency is lower than 150 MHz, there is no further reduction to be carried out, as the spur is too small and exceeds the detection accuracy of the proposed detector.

A comparison of spurs’ characteristics is provided in Table 1, which shows that the proposed design has a better SFDR index than those without auxiliary spurs reduction [19,20]. On the other hand, the method proposed in this paper can achieve a similar SFDR index to that of DDS adopting an auxiliary core but requiring external test equipment [9,10]. The improved SFDR reaches about −60 dBc, and the spur at this time is about −81 dBm, proving the effectiveness of the detector. Table 2 shows the main technical indicators of the detector; its power consumption is obtained by testing the current difference in current before and after enabling the detector. It should be pointed out that the maximum operating frequency is determined only by the frequency of DDS in this paper, not the frequency of this detector, which can be extended according to different applications.

## 5. Conclusions

This study proposed an automatic spurs detector and applied it to detect the clock-induced spurs of DDS. The spurs detector is mainly composed of an energy detector, detection algorithm, and programmable LPF. The programmable LPF with a wide frequency range realizes the coverage of target spurs. Then, the energy detector, based on square operation, will confirm the magnitude of spurs and extract their features with the algorithm. Finally, the extracted features are sent to the auxiliary DDS to reduce the spurs. The detection circuits are applied to a four-channel 1 GSPS DDS and integrated into a chip, which was fabricated with a 65 nm CMOS process with an area of 190 μm × 320 μm. The power consumption is 38 mW. Furthermore, it can be turned off after extracting to save power. The automatic detection ability of the proposed detector for clock-induced-spurs has been proven. The SFDR after reduction is close to that of traditional auxiliary DDS architecture. The automatic detection of spurs without any off-chip auxiliary instruments can greatly improve the convenience and practicability of DDS.

## Figures and Tables

**Table 1:** Comparing the spurs characteristics with other DDSs.

Works	[9]	[10]	[19]	[20]	This Work
Power supply	3.3 V/1.8 V	3.3 V/1.8 V	N/A	2.5 V/1.2 V	2.5 V/1.2 V
Process [nm]	180 CMOS	180 CMOS	55 CMOS	90 CMOS	65 CMOS
Total area [mm^2^]	N/A	19.32 ^(1)^	0.1 ^(1)^	2 ^(1)^	6.9/channel ^(1)^
Structure	LDAC ^(2)^	LDAC ^(2)^	NLDAC ^(2)^	Hybrid ^(2)^	LDA ^(2)^
No. of Channel	1	1	1	1	4
$f_{s}$ [MHz]	1000	2500	2000	1300	1000
Spur reducing	Auxiliary ^(3)^	Auxiliary ^(3)^	N/A	N/A	Automatic detection
SFDR [dB]	−59 ^(4)^	−58 ^(4)^	−55.1	−52	−59.9 ^(4)^
Power	0.7 W@ 1 GHz ^(5)^	1.9 W@ 1 GHz ^(5)^	0.13 W@ 2 GHz ^(5)^	0.35 W@ 1.3 GHz ^(5)^	0.6 W/channel @1 GHz ^(5)^

^(1)^ Including pads. ^(2)^ NLDAC, LDAC and Hybrid are the abbreviations of nonlinear DAC, linear DAC and hybrid DAC respectively. ^(3)^ Spectrum analyzer and other instruments are required. ^(4)^ After reduced. ^(5)^ Total power consumption of DDS.

**Table 2:** Summary of detector parameters and measurements.

Index	This Work
Power of detector [mw]	40 ^(1)^/38 ^(2)^
Area [mm^2^]	0.06
Detection accuracy [dBm]	−82 ^(1)^/−81 ^(2)^
Detection time [μs] ^(3)^	1 ^(1)^
Maximum operating frequency [MHz]	519 ^(1)^/400 ^(2)^

^(1)^ Simulation result. ^(2)^ Test result. ^(3)^ The time needed for one energy detection operation.
